# Iron Chelation Therapy with Deferasirox Results in Improvement of Liver Enzyme Level in Patients with Iron Overload-Associated Liver Dysfunction

**DOI:** 10.1155/2010/675060

**Published:** 2010-05-31

**Authors:** Yasuo Miura, Yusuke Matsui, Hitomi Kaneko, Mitsumasa Watanabe, Mitsuru Tsudo

**Affiliations:** Department of Hematology, Osaka Red Cross Hospital, 5-30 Fudegasaki, Ten-noji, Osaka 543-8555, Japan

## Abstract

Iron chelation therapy (ICT) has been applied for the patients with iron overload-associated liver dysfunction since it is one of the causes of death in patients with intractable hematological diseases requiring multiple red blood cell transfusions. Recently, deferasirox (DSX), a novel, once-daily oral iron chelator, was demonstrated to have similar efficacy to the conventional continuous infusion of deferoxamine on a decrease in serum ferritin (SF) level in heavily transfused patients. We show three cases of transfusion-mediated iron-overloaded patients with an elevated serum alanine aminotransaminase (ALT). All three patients who received the ICT with DSX showed a decrease in ALT level in association with a decrease in SF level. It is suggested that DSX therapy could be considered to expect the improvement of liver damage for iron-overloaded patients with an abnormal ALT level.

## 1. Introduction

Red blood cell (RBC) transfusion therapy is used in the clinical setting for a variety of indications. In the hematological disorders such as myelodysplastic anemia (MDS) or aplastic anemia (AA), RBC transfusions are a life-saving treatment for patients with chronic anemia. As the number of RBC transfusion increases, patients develop iron overload-associated dysfunction in various organs since the human body has no mechanisms to actively excrete excessive iron. Liver is one of the most affected organs since it is the primary site of iron storage in the body, and multiple RBC transfusions result in hepatocellular injury and progression to liver failure. Recent study demonstrated that 6.7% cases of death among patients with the transfusion-dependent anemia were caused by liver failure due to the significantly heavy RBC transfusions [1]. It was reported that the overall survival in transfusion-dependent MDS patients was significantly shorter than that in those who did not require transfusions [2]. Although the direct association between iron overload and short survival, especially in adults with AA or MDS has not been clearly shown, and the association between iron chelation therapy (ICT) and prolonged survival is still questionable, ICT has been applied for the transfusion-dependent patients to expect prevention of iron overload-associated liver dysfunction.

Recently, deferasirox (DSX), a novel, once-daily oral iron chelator, was demonstrated to have similar efficacy to the conventional continuous infusion of deferoxamine (DFO) on a decrease in serum ferritin (SF) level in heavily transfused patients with sickle cell disease [3]. In addition, it was shown in a large EPIC study that there was a trend of decrease in an average serum alanine aminotransaminase (ALT) level among transfusion-dependent MDS patients receiving ICT with DSX [4]. However, it remains fully elucidated whether iron-overloaded individuals with an elevated ALT achieve a decrease in ALT level by the ICT with DSX. In this paper, we show three cases of transfusion-mediated iron-overloaded patients with hematological diseases showing an elevated ALT. The ALT was decreased and subsequently normalized by the treatment with DSX in association with a decrease in SF level in all the three patients.

## 2. Case Presentation

From October 2008 to December 2009, ICT with DSX was initiated in our institute for 19 patients with hematological diseases including MDS (*n* = 9), aplastic anemia (AA, *n* = 7), myeloproliferative neoplasm (MPN, *n* = 2), and congenital hemolytic anemia (*n* = 1). Of these, 3 patients including AA (*n* = 1, UPN #4) and MDS (*n* = 2, UPN #2, #8) were diagnosed as iron overload-associated liver dysfunction. Clinical diagnosis of iron overload-associated liver dysfunction was made as follows: either SF level of above 1000 ng/mL or that of below 1000 ng/mL with a history of multiple RBC transfusions of more than 20 transfusion episodes and an elevated ALT level above 44 IU/L. All patients received serological tests for antihepatitis C virus (HCV) antibody and antihepatitis B surface antigen (HBsAg) and abdominal ultrasound to show no other apparent causes for an elevated ALT level including infection of HBV and HCV, liver mass. In addition, drug- or alcohol-induced liver injury was excluded based on the present illness. Normalization of liver enzyme level was defined as ALT below 44 IU/L on consecutive two laboratory assessments. All the patients had no other apparent factors influencing the SF levels. 

A 33-year-old female with AA (UPN #4) received transfusion of more than 222 packed RBC units (1 packed RBC unit derived from 200 mL whole blood) for 10 years in combination with intermittent administration of DFO (21 g per year). Her SF value was kept high around 10000 ng/mL. Eight years after the onset of AA, she developed diabetes mellitus requiring insulin therapy. Laboratory examinations at baseline showed that ALT and SF were elevated to 42 IU/L and 9291 ng/mL, respectively. She received DSX therapy at a dose of 1000 mg/day (16.1 mg/kg/day). The ALT was gradually decreased accompanied with a decrease in SF level. At seven months post DSX therapy, the ALT was normalized to 42 IU/L when the SF was decreased to 5147 ng/mL. Both aspartate aminotransaminase (AST) and alkaline phosphatase (ALP) were also elevated to 85 IU/L (upper normal limit: 38 IU/L) and 818 IU/L (upper normal limit: 338 IU/L), respectively before ICT. At seven months post DSX therapy, AST was normalized to 35 IU/L and ALP decreased to 519 IU/L. Total bilirubin (T-Bil) was within normal limit before and after ICT and showed almost no change.

A 74-year-old male with MDS (UPN #8) received transfusions of 42 packed RBC units prior to DSX therapy and required chronic transfusions of 8 packed RBC units per month. Laboratory examinations showed that ALT and SF were elevated to 72 IU/L and 886.8 ng/mL, respectively. DSX treatment with an initial dose of 1000 mg/day (16.7 mg/kg/day) was applied, which was followed by a reduction of DSX dose to 500 mg/day (8.4 mg/kg/day) due to an elevated serum creatinine from 1.00 mg/dL to 1.49 mg/dL. The ALT reached normal level (44 IU/L) at 5 weeks post DSX therapy. At the same point, SF was decreased to 485.9 ng/mL. The average dose of DSX was 14.9 mg/kg/day. The AST was high at 90 IU/L at the baseline, and it was decreased to 55 IU/L at 5 weeks post DSX therapy. The *γ*-glutamyl transpeptidase (*γ*-GTP) and T-Bil were high at 99 IU/L and 1.9 mg/dL, respectively, before ICT, and these parameters showed almost no change post DSX therapy. 

A 56-year-old female with MDS complicated with nephrotic syndrome (UPN #2) received transfusions of 82 packed RBC units before she received DSX therapy. The transfusion requirement was 2.7 packed RBC units in a month during DSX therapy. Laboratory examinations showed that ALT and SF were elevated to 84 IU/L and 1436 ng/mL, respectively. She received 500 mg/day (10.6 mg/kg/day) of DSX for the first 3 weeks and received 1000 mg/day (21.3 mg/kg/day) of DSX thereafter. At 15 weeks post DSX therapy, it was confirmed that the ALT value reached normal level at 31 IU/L when the SF was decreased to 994.8 ng/mL. Immunosuppressive therapy with cyclosporine had been applied to this patient for the treatment of MDS. After the administration of DSX, serum creatinine was elevated to the maximal of 1.64 mg/dL from the baseline level of 0.95 mg/dL. Reduction in cyclosporine dosage by 33% brought about improvement of creatinine level to 1.18 mg/dL in the absence of the adjustment of DSX dosage. The average dose of DSX was 19.1 mg/kg/day. The AST was high at 64 IU/L before ICT, and it was normalized to 36 IU/L at 15 weeks post DSX therapy. The ALP, albumin (Alb), and T-Bil were normal before ICT, and these parameters showed almost no change post DSX therapy.

## 3. Discussion

In this paper, we show three cases of transfusion-mediated iron-overloaded patients with an elevated ALT, whose level was decreased by the ICT with DSX. This favorable effect of DSX on abnormal ALT level was observed in all patients who received the ICT during the observation periods (three out of three patients). The abnormal AST level was also decreased in all 3 patients receiving DSX therapy. The other parameters regarding the liver function including ALP, *γ*-GTP, T-Bil, and Alb seemed not to be apparently changed. Our cases support the observation in a large EPIC study that the ICT with DSX exhibited a trend of decrease in an average ALT among the transfusion-dependent patients. In addition, our observation provides additional information that not only individual patients with normal ALT but also those with an elevated abnormal ALT could get benefit of improving the liver dysfunction from the ICT with DSX.

We did not have possibility to perform a liver biopsy to examine histology in our patients because all patients were thrombocytopenic and had risk of bleeding complication caused by a liver biopsy. Therefore, the association between liver damage and iron overload is circumstantial and not directly proven, and it was unclear whether the improvement of abnormal ALT accompanied with a decrease in SF was associated with the improvement of histological damage in liver. Regarding this issue, previous study showed that hepatic fibrosis occurs at around 400 uM/g of liver iron concentrations (LICs) in patients with hereditary hemochromatosis [5]. It was also demonstrated in patients with transfusional iron overload that elevation of ALT was observed only when LIC exceeded 300 to 400 uM/g and that abnormal ALT level became normal when LIC fell below 350 uM/g with iron chelation therapy with DFO [6]. These observations imply the existence of critical LIC of 300 to 400 uM/g above which histological hepatocellular injury is developed and is indicated by an elevated ALT. Therefore, we would suggest that a decrease in ALT by the ICT with DSX might be one of the parameters reflecting the improving liver damage in iron-overloaded patients.

In summary, three cases of transfusion-mediated iron-overloaded patients with hematological diseases exhibiting improvement of abnormal ALT by the ICT with DSX were presented. It was suggested that DSX therapy could be considered for such patients to expect the improvement of liver damage.
